# Clinical and Mycological Profiles of Chronic and Recalcitrant Dermatophytosis: Türkiye, 2022–2024

**DOI:** 10.1007/s11046-026-01058-5

**Published:** 2026-02-18

**Authors:** Murat Durdu, Hazal Kandemir, Ayşe Sultan Karakoyun, Pavlína Lysková, Daniela Kolarczyková, Oğuzhan Bingöl, Yasemin Erdem, Funda Aktürk-Gözel, Özlem Su-Küçük, Melisa Özay, Aslan Yürekli, İlayda Muslu, İsa An, Sinan Özçelik, Tuğba Falay-Gür, Tuğba Tehçi, Abdullah Demirbaş, Gözde Ulutaş-Demirbaş, Tuğba Atak, Kaan Taşolar, Büşra Acar-Mantar, Mehmet Kamil Mülayim, Rebiay Kıran, Ayşe Tülin Mansur, İrem Yanatma, Sertaç Sever, Yeşim Yayla-Öztürk, Selami Aykut Temiz, Rabia Öztaş-Kara, Fatma Uzun, Ferit Kuşçu, Gonca Erköse-Genç, Zayre Erturan, Sybren de Hoog, Vit Hubka, Macit Ilkit

**Affiliations:** 1 Department of Dermatology, Dr. Turgut Noyan Application and Research Center, Başkent University, Adana, Turkey; 2 Westerdijk Fungal Biodiversity Institute, Utrecht, the Netherlands; 3 Department of Environmental Biology, Institute of Environmental Sciences (CML), Leiden University, Leiden, the Netherlands; 4 Division of Mycology, Department of Microbiology, Faculty of Medicine, University of Çukurova, Adana, Turkey; 5 Department of Medical Microbiology Prague and Kladno, Public Health Institute in Ústí Nad Labem, Prague, Czech Republic; 6 Department of Botany, Faculty of Science, Charles University, Prague, Czech Republic; 7 Laboratory of Fungal Genetics and Metabolism, Institute of Microbiology, Czech Academy of Sciences, Prague, Czech Republic; 8 Department of Dermatology and Venereology, İstanbul Faculty of Medicine, İstanbul University, Istanbul, Turkey; 9 Department of Dermatology and Venereology, School of Medicine, Bezmialem Vakıf University, Istanbul, Turkey; 10 Training and Research Hospital Dermatology Department, Muğla Sıtkı Koçman University, Muğla, Turkey; 11 Department of Dermatology, Şanlıurfa Training and Research Hospital, Şanlıurfa, Turkey; 12 Private Dermatology Clinic, Istanbul, Turkey; 13 Department of Dermatology, University of Health Sciences -Adana Health Practice and Research Center, Adana, Turkey; 14 Department of Dermatology, Faculty of Medicine, Kocaeli University, Kocaeli, Turkey; 15 Department of Dermatology, Kocaeli City Hospital, Kocaeli, Turkey; 16 Ministry of Health Islahiye State Hospital, Dermatology Clinic, Gaziantep, Turkey; 17 Private Sular Academy Hospital, Dermatology Clinic, Kahramanmaraş, Turkey; 18 Bartın State Hospital, Dermatology Clinic, Bartın, Turkey; 19 Faculty of Medicine Department of Dermatology, Sütçü İmam University, Kahramanmaraş, Turkey; 20 Academic Hospital, Dermatology Clinic, Istanbul, Turkey; 21 Fethiye State Hospital, Dermatology Clinic, Muğla, Turkey; 22 Department of Dermatology, Avicenna Umut Hospital, Istanbul, Turkey; 23 Private Olimpos Hospital, Dermatology Clinic, Antalya, Turkey; 24 Department of Dermatology, Necmettin Erbakan University Medical Faculty, Konya, Turkey; 25 Sakarya Education and Research Hospital, Dermatology Clinic, Sakarya, Turkey; 26 Gemlik State Hospital, Dermatology Clinic, Bursa, Turkey; 27 Department of Infectious Diseases and Clinical Microbiology, Faculty of Medicine, University of Çukurova, Adana, Turkey; 28 Department of Medical Microbiology, İstanbul Faculty of Medicine, İstanbul University, Istanbul, Turkey; 29 Center of Expertise in Mycology of Radboud University Medical Center/Canisius Wilhelmina Hospital, Nijmegen, the Netherlands; 30 Foundation Atlas of Clinical Fungi, Hilversum, the Netherlands

**Keywords:** Antifungal resistance, Curcumin, Dermatophytosis, *SQLE*, Resveratrol, *Trichophyton indotineae*

## Abstract

This study investigated the treatment outcomes of 91 patients with chronic and recalcitrant dermatophytosis from 20 hospitals in Türkiye between June 2022 and March 2024, as well as the in vitro antifungal susceptibility and squalene epoxidase (*SQLE*) substitution profiles of the dermatophytes obtained from these patients. Molecular identification by sequencing 91 isolates, revealed 76 *Trichophyton mentagrophytes* ITS genotype VIII (TmVIII; also known as *T*. *indotineae*) isolates, 13 *Trichophyton rubrum*, one *Trichophyton tonsurans*, and one *Microsporum canis*. The Phe397Leu substitution predominated in the *SQLE*, and Tyr414His co-occurred in nine TmVIII isolates. Five novel substitutions were observed in TmVIII. Among 13 *T*. *rubrum* isolates, three showed different *SQLE* substitution patterns compared to the wild-type strain. EUCAST antifungal susceptibility testing revealed high minimum inhibitory concentrations (MICs) for terbinafine (TRB) (> 0.25 mg/L) and for fluconazole (FLZ) (≥ 16 mg/L), indicating resistance in most TmVIII isolates. Of the 91 patients, 83 responded to FLZ, itraconazole (ITZ), TRB, or voriconazole-based regimens. While adjunct therapy with FLZ and resveratrol (RES) led to improvement in 28/29 patients despite their high in vitro inhibition concentrations, ITZ and curcumin (CUR) therapy was successful in 3/3 patients. However, the in vitro efficiency of CUR could not be evaluated. These findings highlight the local occurrence of specific *SQLE* substitutions, underscore the importance of EUCAST MIC testing combined with *SQLE* sequencing for surveillance, and support further evaluation of RES and CUR as an adjunct in TRB-resistant TmVIII.

**Supplementary Information:**

The online version contains supplementary material available at 10.1007/s11046-026-01058-5.

## Introduction

*Trichophyton* species are the most common causative agents of dermatophytoses worldwide. The genus comprises strictly anthropophilic species, such as *T. rubrum*, *T. tonsurans*, and *T. violaceum*, as well as zoophilic species including *T. benhamiae*, *T. equinum*, and *T. mentagrophytes* [1]. The classification of *Trichophyton* species has been challenging due to the difficulty in differentiating closely related species. The use of multiphasic approaches, i.e., integrating host specificity, virulence, growth characteristics, sporulation, microscopic features, metabolite production, and mating behavior with molecular data, has significantly improved dermatophyte species definition and identification. However, due to the complexity of distinguishing closely related species, dermatophytes are often grouped into “species complexes”, referred to as “varieties” or named after their internal transcribed spacer (ITS) genotypes [1–4]. Based on recent developments in the taxonomy of *Trichophyton* [5–7], *T*. *indotineae* is also accepted as the ITS genotype VIII or variety *indotineae* within the *T*. *mentagrophytes* complex; however, the name *T*. *indotineae* is commonly used in clinical applications for practical reasons.

TmVIII has been reported in at least 49 countries across six continents [7, 8] and is drawing considerable attention due to its frequent resistance to terbinafine (TRB), commonly used first-line treatment for dermatophytoses worldwide [9]. A large proportion of TmVIII isolates reported from various countries are TRB-resistant, with one or multiple specific substitutions in their squalene epoxidase (*SQLE*) gene linked to resistance [8].

Although still less common than TmVIII, TRB resistance can also be detected in other dermatophytes driven by similar *SQLE* gene mutations [5, 6, 10, 11]. In such cases, azole-class antifungals are preferred for treatment. However, azole antifungals can pose challenges due to their potential for drug-drug interactions, particularly in patients on complex medication regimens [12]. In such cases, other safe and effective treatment options are urgently needed. Resveratrol (RES), a polyphenolic antioxidant found in red wine, grapes, and peanuts, has been shown to inhibit the growth of dermatophytes in vitro [13]. Other natural compounds, namely curcumin (CUR), derived from the rhizome of *Curcuma longa* plants, and turmeric oil, a product of CUR, have also demonstrated antifungal activity against dermatophytes in both in vitro and in vivo studies [14, 15]. Nevertheless, the susceptibility profile of TRB-resistant dermatophytes to these compounds has not been investigated, and clinical data supporting their therapeutic efficacy remain limited [14–17].

Recently, Durdu et al. [17] reported the first cases of TRB-resistant TmVIII from a hospital in Türkiye and successfully treated one of these patients with RES tablets without the need for further antifungal treatment. These cases formed the basis of the present study, which explores the resistance profile of isolates obtained from patients with chronic and recalcitrant dermatophytosis in various locations in Türkiye, along with patient characteristics and treatment responses to available antifungal compounds, including RES and CUR.

## Materials and Methods

### Patients

**Fig. 1:**
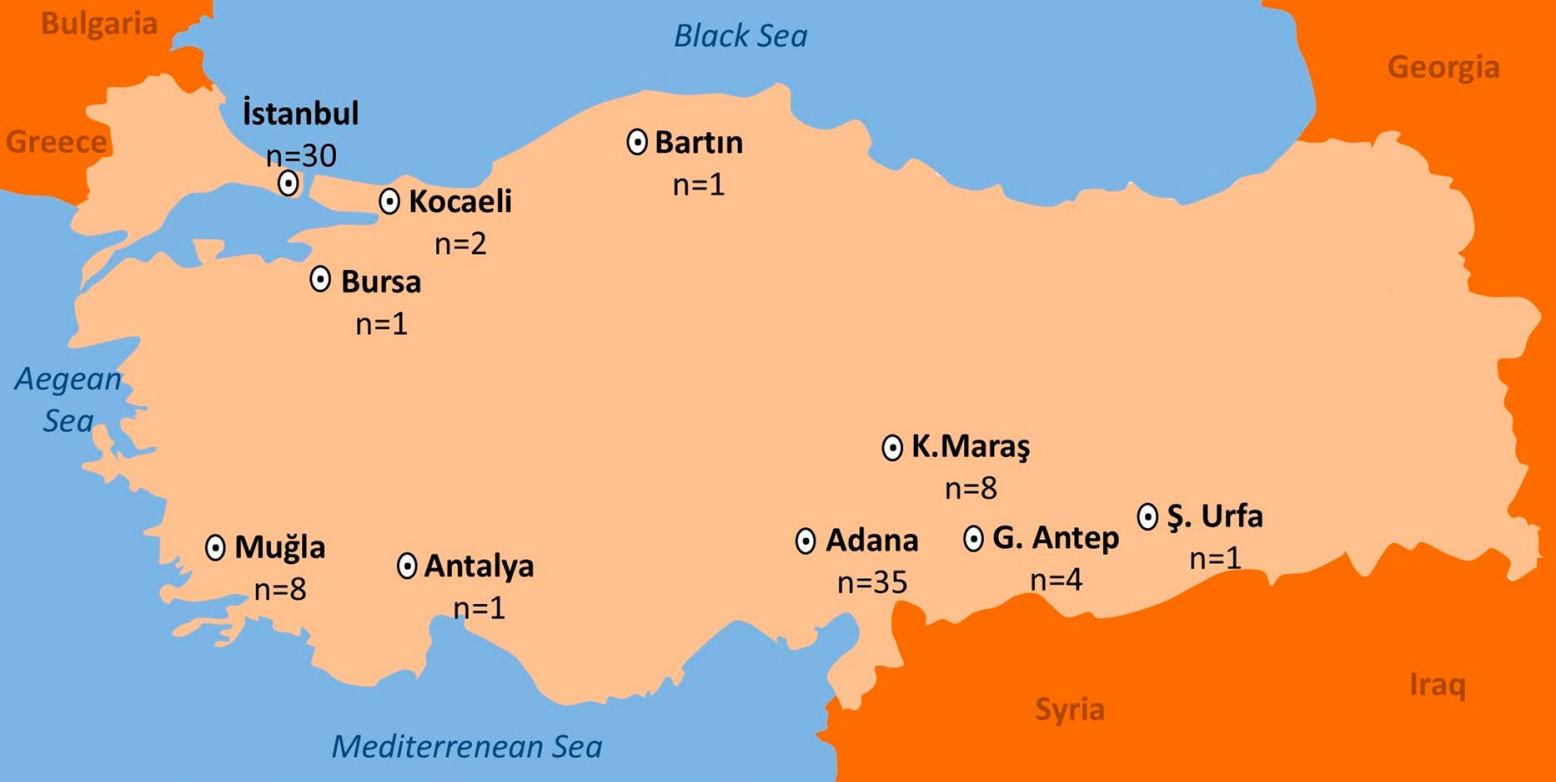
Geographical location and the number of skin samples collected from 20 hospitals across 10 cities in Türkiye that participated in this study. Detailed information for the participant hospitals and the sample size from each hospital has been provided in the Table S1

A total of 91 patients with chronic and recalcitrant dermatophytosis between June 2022 and March 2024 were enrolled from 20 hospitals across 10 cities in Türkiye (Fig. 1). Patients presented with erythematous, scaly patches and/or plaques on their bodies, diagnosed with dermatophyte infection on microscopic examination and/or cultivation, and whose symptoms persisted despite treatment with topical and systemic antifungals for at least 4 weeks were included in the study. Data were collected on patients’ age, sex, medical history, duration, and anatomical location of infection, and the presence of similar symptoms in household members. Informed consent was obtained from all participants or legal guardians for those under 18 years of age. Each hospital provided an approval letter to participate in the study, and the final ethical statement was issued by the Bezmialem Vakıf University Rectorate Technology Transfer Office Ethics Committees Unit, Istanbul, Türkiye, with the approval number: 2024/107.

### Isolates

Skin scraping samples were collected and inoculated onto Sabouraud glucose agar (SGA; Merck, Darmstadt, Germany) supplemented with 100 µg/mL chloramphenicol (Sigma-Aldrich, Steinheim, Germany) and 50 µg/mL gentamicin (Sigma-Aldrich). The plates were incubated at 28 °C for up to 14 days at each hospital, and the isolates were sent to Charles University and the Westerdijk Fungal Biodiversity Institute for further molecular and antifungal susceptibility testing.

DNA was extracted from fresh colonies using the Wizard® Genomic DNA Purification Kit (Promega Corp., Madison, WI, USA). Molecular identification was performed by sequencing the nuclear ribosomal internal transcribed spacer region (ITS), the D1–D2 region of the large subunit (LSU), partial β-tubulin (*tubb*), and translation elongation factor 1-α (*tef-1α*) loci using the primers V9G/LS266 [18], LR0R-LR5 [19], T1/tub4rd [20, 21], and EF1/EF2 [22, 23] respectively. The partial *SQLE* gene was amplified and sequenced using primers described by Kong et al. [24] to detect amino acid substitutions. The PCR amplicons were visualized on a 1.5% agarose gel, and sequencing was performed using Applied Biosystems BigDye Terminator version 3.1 (Thermo Fisher Scientific). The sequences were assembled and edited using Geneious R11 [25] and aligned with MAFFT v.7 [26]. After the best-fit model for each gene was determined using ModelFinder [27] based on the Bayesian Information Criterion (BIC), phylogenetic analyses were conducted using the maximum likelihood method with ultrafast bootstrap option (1000 iterations) implemented on the IQ-TREE web server (http://www.iqtree.cibiv.univie.ac.t/) [28–30]. Trees were visualized using FigTree v1.4.4 [31] and the online Interactive Tree of Life (iToL) server v6 [32]. Reference *SQLE* sequences MW187977.1 (wild-type of TmVIII) and OM313296.1 (wild-type of *T*. *rubrum*) were used for detecting substitutions. The GenBank accession numbers of all sequences used in this study are listed in the Table S2.

### Antifungal Susceptibility Testing

The broth microdilution method was performed following the EUCAST guidelines (E.Def 11.0) [33]. The antifungals (Sigma-Aldrich, Prague, Czech Republic) were dissolved in dimethyl sulfoxide (DMSO, Sigma Aldrich), and their test concentration ranges (mg/L) were as follows: fluconazole (FLZ; 0.06–64), terbinafine (TRB; 0.004–4), itraconazole (ITZ; 0.008–8), posaconazole (PCZ; 0.008–8), voriconazole (VRZ; 0.008–8), ketoconazole (KCZ; 0.008-8), and resveratrol (RES; 0.5–512). The isolates were subcultured at 25 °C on SGA (Trios, Prague, Czech Republic). Inoculum suspensions were prepared from 1- to 3-week-old colonies to achieve sufficient sporulation. The suspensions were prepared in sterile distilled water supplemented with 0.1% Tween 20 and filtered through sterile nylon filters (11 μm pore size; Merck, Prague, Czech Republic). To adjust the density of the suspension to 0.5 McFarland (concentration of 2–5 × 10^6^ conidia/mL), distilled water was added as needed, and density was verified using a DENSI-LA-METRB II densitometer (Erba Lachema, Brno, Czech Republic). The suspension was then diluted 1:10 with sterile distilled water to obtain a final working inoculum of 2–5 × 10^5^ conidia/mL. The inoculum suspension was supplemented with chloramphenicol (final concentration 50 mg/L; Sigma-Aldrich, St. Louis, USA) and cycloheximide (final concentration 300 mg/L; Sigma-Aldrich). The microplates were incubated at 25 °C in ambient air for 5–7 days. The MIC endpoints were read spectrophotometrically as 50% growth inhibition. The endpoints for RES were visually interpreted as complete inhibition of fungal growth. *Candida krusei* (ATCC 6258), *C*. *parapsilosis* (ATCC 22019), *Aspergillus flavus* (ATCC 204304), and *A*. *flavus* (CNM-1813) were used as quality control strains. In this study, CUR (C1386, Sigma-Aldrich) dissolved poorly in both water and dimethyl sulfoxide and was excluded from in vitro antifungal susceptibility testing.

### Statistical Analysis

The Chi-square test was used in large samples (expected value ≥ 5 in each cell) to compare the frequency of dermatophyte infections between females and males across different anatomical regions, and Fisher’s exact test was used when any expected cell count was < 5. The significance threshold was set at p < 0.05. All statistical analyses were performed using the IBM SPSS Statistics v 21.0 (IBM Corp., Armonk, NY, USA).

## Results

### Patients

**Fig. 2:**
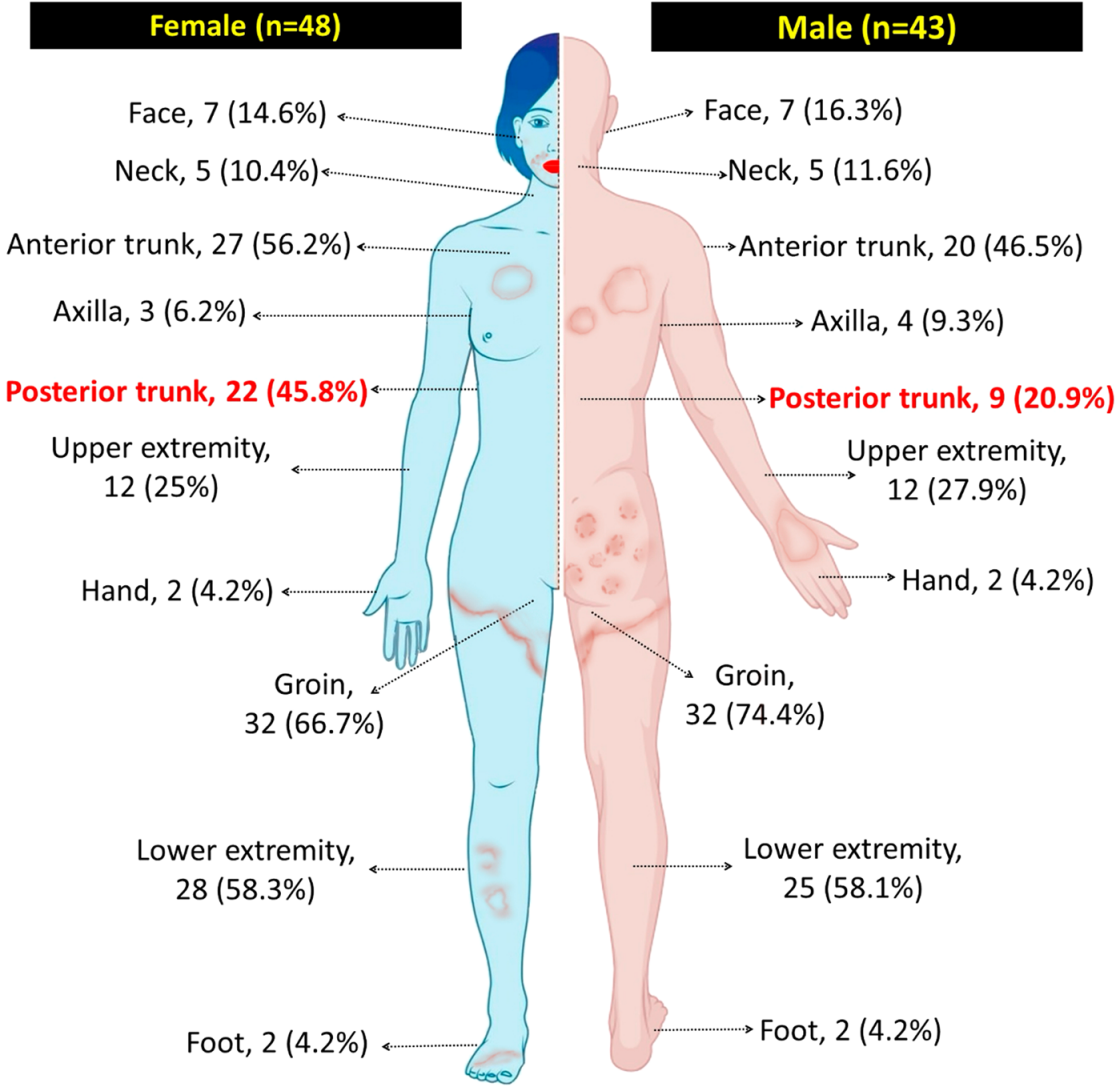
Distribution of the lesions among male and female patients

Ninety-one patients with microscopically confirmed dermatophytosis and a history of prior treatment failure, identified across 20 hospitals in 10 cities in Türkiye, were included in this study (Fig. 1). The majority of cases were reported from Adana (*n* = 36) and Istanbul (*n* = 30). Of the 91 patients, 43 (47.3%) were male and 48 (52.7%) were female, with ages ranging from 4 to 69 years (median age = 35 years). The duration of infection ranged from 4 to 36 months, with a median of 11 months. One participant was pregnant, and 18 had preexisting conditions such as diabetes (*n* = 6), hypertension (*n* = 4), asthma (*n* = 4), HIV (*n* = 1), kidney transplantation (*n* = 1), STAT3 mutation (*n* = 1), or sickle cell anemia (*n* = 1). None of the patients had a history of travel to India or the surrounding areas. One patient was identified as a sex worker. One patient with asthma also had epilepsy. Thirty-two patients (35.2%) indicated that at least one family member was also affected, and this concerned mostly the partner (*n* = 15). Lesions were found in multiple anatomical locations in 75 patients (82.4%). The most frequently affected areas were the groin and gluteal regions (*n* = 64; 70.3%), followed by the lower extremities (*n* = 53; 58.2%), anterior trunk (*n* = 47; 51.6%), upper extremities (*n* = 35; 38.5%), posterior trunk (*n* = 31; 34.1%), and face (*n* = 16; 17.6%). Posterior trunk involvement was significantly more common in females (*n* = 22; 45.8%) than in males (*n* = 9; 20.9%) (*p* = 0.027) (Fig. 2). Only two patients reported no itching; in the remaining cases, pruritus was the most frequently reported symptom (*n* = 89). Dermatophytide (id) reactions, one with a maculopapular eruption and the other with dyshidrotic eczema, were observed in two patients with no underlying disease.

### Isolates

**Fig. 3:**
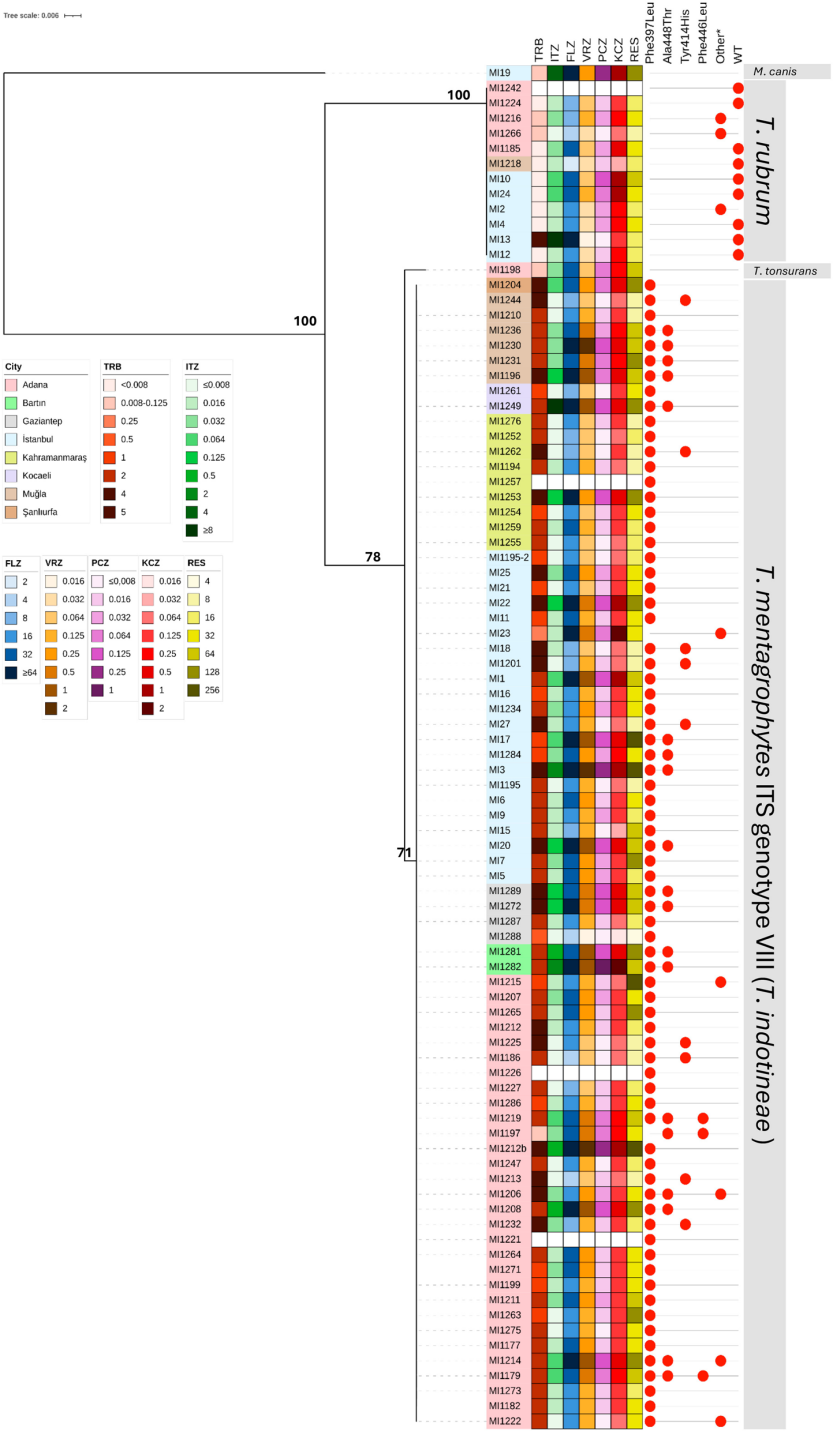
Phylogenetic tree based on ITS sequences, combined with the location, *SQLE* substitutions, and antifungal susceptibility test results of 90 isolates. The phylogenetic tree was constructed using the IQ-Tree web server, based on the best model TN + F + G4 (BIC). The alignment consists of 965 bp, 66 parsimony-informative, and 798 constant sites. Values of maximum likelihood are shown on the branches. Antifungal susceptibility test could not be performed with the isolates MI1221, MI1226, MI1242, and MI1257 due to contamination. *See Table 1

Among the 91 isolates from treatment-refractory dermatophytosis, 76 were identified as TmVIII, 13 as *T*. *rubrum*, one as *T*. *tonsurans*, and one as *Microsporum canis* based on ITS, *tubb*, and *tef-1α* sequencing results (Fig. 3, Fig. S1). In total, 74/76 TmVIII isolates (97%) exhibited the Phe397Leu amino acid substitution in the partial *SQLE* gene, and in nine of these 74 isolates, Tyr414His co-occurred. In addition to Phe397Leu and Tyr414His, we observed the previously described substitutions Ala448Thr and Leu393Ser [24, 34, 35], along with several novel substitutions, that co-occurred with Phe397Leu (Phe446Leu, Phe446Gly, Phe446Cys, Phe446Val, and Val436Gly) (Table 1).

**Table 1:** Clinical features of the patients, including treatment history and *SQLE* substitutions in dermatophyte isolates

Patient no	Gender	Age (year)	Location of the lesions	Duration of the infection (month)	Similar complaints in the family	Co-existing conditions	Previous treatment^a^	Successful treatment^a^	Species/Strain	*SQLE* substitutions^b^
1	F	42	Trunk-front and back, face, groin and gluteal regions, foot	24	–	STAT mutation	TRB 250 mg/d for 2 m, ITZ 200 mg/d for 3 m	FLZ 200 mg/d for 2 m with RES	TmVIII/MI1177	Phe397Leu
2	F	41	Upper and lower extremities, trunk-back	12	Partner	–	TRB 3 m, ITZ 100 mg/d for 3 m	FLZ 200 mg/d for 2 m with RES	TmVIII/MI1179	Phe397Leu **Phe446Leu** Ala448Thr
3	M	31	Trunk-front and back, upper and lower extremities, groin and gluteal regions	12	–	–	TRB 250 mg/d for 2 m, ITZ 200 mg/d for 2 m	FLZ 200 mg/d for 2 m with RES	TmVIII/MI1182	Phe397Leu
4	M	39	Groin and gluteal regions	24	–	–	TRB 250 mg/d for 2 m, ITZ 100 mg/d for 2 m, FLZ 150 mg/w 3 m	FLZ 200 mg/d for 2 m with RES	TmVIII/MI1186	Phe397Leu Tyr414His
5	M	64	Lower extremities, trunk-front, groin and gluteal regions	6	–	Diabetes mellitus	TRB 250 mg/d for 6 m	ITZ 200 mg/d for 1 m with CUR food supplement	TmVIII/MI1194	Phe397Leu
6	M	31	Trunk-back, armpit, face, groin and gluteal regions	11	–	–	TRB 250 mg/d for 4 m	ITZ 200 mg/d for 2 m^c^	TmVIII/MI1195	Phe397Leu
7	F	39	Upper and lower extremities, trunk-front, face	18	Partner	–	TRB 250 mg/d, ITZ 200 mg/d for 3 m	FLZ 200 mg/d for 3 m^c^	TmVIII/MI1196	Phe397Leu Ala448Thr
8	F	62	Trunk-front and back, upper extremities, groin and gluteal regions	5	–	–	ITZ 100 mg/d for 2 m, FLZ 150 mg/w for 1 m	TRB 250 mg/d for 2 m	TmVIII/MI1197	**Phe446Leu** Ala448Thr
9	F	30	Lower extremities	24	–	–	TRB 250 mg/d and ITZ 200 mg/d for 3 m	FLZ 200 mg/d 2 m with RES	TmVIII/MI1199	Phe397Leu
10	M	49	Face, groin and gluteal regions, trunk-back, neck, upper and lower extremities	6	–	Allergic asthma, epilepsy	TRB 250 mg/d for 1 m, FLZ 200 mg/d for 1 m, ITZ 100 mg/d for 7 m, ITZ 200 mg/d for 2 m	ITZ 200 mg/d for 2 m^d^	TmVIII/MI1201	Phe397Leu Tyr414His
11	M	20	Groin and gluteal regions	7	Partner	–	TRB 250 mg/d for 2 m, ITZ 100 mg/d for 1 m	ITZ 400 mg/d 2 m	TmVIII/MI1204	Phe397Leu
12	M	47	Lower extremities, trunk-front, groin and gluteal regions	36	Partner	–	TRB 250 mg/d for 3 m, ITZ 200 mg/d for 4 m, FLZ 200 mg/d for 3 m	None	TmVIII/MI1206	Phe397Leu **Phe446Gly** Ala448Thr
13	F	26	Trunk-back, lower extremities, groin and gluteal regions	12	Partner	–	TRB 250 mg/d, ITZ 200 mg/d for 3 m	FLZ 200 mg/d for 2 m with RES	TmVIII/MI1207	Phe397Leu
14	M	35	Upper and lower extremities^e^	12	Partner	–	TRB 250 mg/d for 1 m	ITZ 100 mg/d for 1 m	TmVIII/MI1208	Phe397Leu Ala448Thr
15	M	28	Trunk-front, groin and gluteal regions	13	–	–	TRB 250 mg/d, ITZ 200 mg/d for 3 m	FLZ 200 mg/d for 3 m with RES	TmVIII/MI1210	Phe397Leu
16	M	45	Groin and gluteal regions	18	Partner	–	TRB 250 mg/d for 2 m, ITZ 200 mg/d for 3 m, FLZ 200 mg/d for 3 m	None	TmVIII/MI1211	Phe397Leu
17	M	31	Groin and gluteal regions, upper and lower extremities	36	Partner	–	TRB 250 mg/d for 8 m, ITZ 200 mg/d for 3 m	None	TmVIII/MI1212A	Phe397Leu
18	F	29	Groin and gluteal regions, upper and lower extremities	36	Partner	–	TRB 250 mg/d for 5 m, ITZ 200 mg/d for 3 m	None	TmVIII/MI1212B	Phe397Leu
19	M	23	Upper and lower extremities, trunk-front, groin and gluteal regions, face, neck	9	Partner		ITZ 100 mg/d for 2 m	FLZ 200 mg/d for 1.5 m with RES	TmVIII/MI1213	Phe397Leu Tyr414His
20	F	46	Face, groin and gluteal regions, armpit, trunk-back	36	Partner	–	TRB 250 mg/d for 4 m, ITZ 200 mg/d for 4 m, FLZ 200 mg/d for 3 m	None	TmVIII/MI1214	Phe397Leu **Phe446Cys** Ala448Thr
21	F	4	Groin and gluteal regions, upper extremities	6	–	–	TRB 125 mg/2 m	ITZ 50 mg/d for 2 m	TmVIII/MI1215	Phe397Leu **Phe446Val**
22	F	18	Groin and gluteal regions, upper and lower extremities, trunk-front and back	9	Sibling	–	TRB 250 mg/d for 3 m	FLZ 200 mg/d for 2 m	TmVIII/MI1219	Phe397Leu **Phe446Leu** Ala448Thr
23	M	22	Groin and gluteal regions	7	–	–	TRB 250 mg/d for 4 m, ITZ 200 mg/d for 7 m	FLZ 200 mg/d for 2 m with RES	TmVIII/MI1195-2	Phe397Leu
24	M	23	Groin and gluteal regions, lower extremities	8	–	–	TRB 250 mg/d for 1 m, ITZ 200 mg/d for 1 m, FLZ 200 mg/d for 2 m	None	TmVIII/MI1221	Phe397Leu
25	F	25	Groin and gluteal regions, upper and lower extremities	10	Parents	Sickle cell anemia	TRB 250 mg/d for 3 m	ITZ 100 mg/d for 1 m	TmVIII/MI1222	Phe397Leu **Val436Gly**
26	M	21	Groin and gluteal regions, trunk-front	8	–	HIV	TRB 250 mg/d for 2 m, ITZ 200 mg/d 1 m	FLZ 200 mg/d for 2 m with RES	TmVIII/MI1225	Phe397Leu Tyr414His
27	F	26	Groin and gluteal regions	8	Colleague	–	TRB 250 mg/d for 2 m	FLZ 200 mg/d for 2 m with RES	TmVIII/MI1226	Phe397Leu
28	F	37	Groin and gluteal regions	6	–	–	TRB 250 mg/d for 1 m	FLZ 200 mg/d for 2 m with RES	TmVIII/MI1227	Phe397Leu
29	M	45	Groin and gluteal regions, lower extremities, armpit, trunk-front	4	–	–	ITZ 200 mg/d for 4 m	FLZ 200 mg/d for 1 m with RES	TmVIII/MI1230	Phe397Leu Ala448Thr
30	F	36	Face, trunk-front, lower extremities	9	–	–	TRB 250 mg for 2 m, ITZ 200 mg for 2 m, FLZ 150 mg/w, for 3 m	FLZ 200 mg/d for 1 m with RES	TmVIII/MI1231	Phe397Leu Ala448Thr
31	M	34	Groin and gluteal regions	12	–	–	TRB 250 mg/d for 2 m, FLZ 200 mg/d for 2 m	ITZ 200 mg/d 2 m	TmVIII/MI1232	Phe397Leu Tyr414His
32	M	19	Groin and gluteal regions, lower extremities, trunk-front	9	–	–	TRB 250 mg/d for 1 m, ITZ 100 mg/d for 1 m	FLZ 200 mg/d for 4 m with RES	TmVIII/MI1234	Phe397Leu
33	F	36	Face, lower extremities, trunk-front	9	–	–	TRB 250 mg for 2 m, ITZ 200 mg for 1 m, FLZ 150 mg/w for 3 m	FLZ 200 mg/d for 1 m with RES	TmVIII/MI1236	Phe397Leu Ala448Thr
34	M	41	Groin and gluteal regions, trunk-front and back	12	Partner	–	TRB 250 mg/d for 2 m	FLZ 200 mg/d for 4 m with RES	TmVIII/MI1244	Phe397Leu Tyr414His
35	F	58	Groin and gluteal regions, trunk-front and back	9	–	–	TRB 250 mg/d for 3 m, ITZ 100 mg/d for 3 m	ITZ 200 mg/d for 4 m	TmVIII/MI1247	Phe397Leu
36	F	52	Trunk-front and back^f^	9	–	–	TRB 250 mg/d for 2 m	FLZ 200 mg/d for 3 m with RES	TmVIII/MI1249	Phe397Leu Ala448Thr
37	M	4	Trunk-back, lower extremities	12	Parents and sibling	–	TRB 250 mg/d for 1 yr	ITZ 100 mg/d for 1 m	TmVIII/MI1252	Phe397Leu
38	M	5	Trunk-front and back, lower extremities	12	Parents and sibling	–	TRB 250 mg/d for 1 yr	ITZ 100 mg/d for 1 m	TmVIII/MI1253	Phe397Leu
39	M	35	Trunk-front and back, lower extremities, groin and gluteal regions	12	Parents and sibling	–	TRB 250 mg/d for 1y, FLZ 150 mg/w for 2 m	ITZ 200 mg/d for 1 m	TmVIII/MI1254	Phe397Leu
40	F	30	Trunk-front and back, upper and lower extremities, groin and gluteal regions	5	Parents and sibling	Diabetes mellitus	Unprescribed topical antifungals	Topical treatments	TmVIII/MI1255	Phe397Leu
41	M	29	Upper and lower extremities, groin and gluteal regions	9	–	–	TRB 250 mg/d for 3 m	ITZ 200 mg/d and CUR food supplement for 1 m	TmVIII/MI1257	Phe397Leu
42	F	22	Lower extremities	6	–	Asthma	TRB 250 mg/d for 1 m	ITZ 200 mg/d and CUR food supplement for 1 m	TmVIII/MI1259	Phe397Leu
43	F	48	Trunk-front and back, upper and lower extremities, groin and gluteal regions, neck	14	Parents and sibling	–	TRB 250 mg/d for 6 m	ITZ 200 mg/d for 3 m	TmVIII/MI1261	Phe397Leu
44	F	51	Trunk-front and back	10	–	Diabetes mellitus, hypertension	TRB 250 mg/d for 3 m	ITZ 200 mg/d for 6 m, FLZ 200 mg/day for 1 m	TmVIII/MI1262	Phe397Leu Tyr414His
45	F	45	Trunk-front and back, upper and lower extremities, groin and gluteal regions	11	–	–	ITZ 200 mg/d for 1 m	FLZ 200 mg/d for 2 m with RES	TmVIII/MI1263	Phe397Leu
46	M	22	Upper extremities, groin and gluteal regions, lower extremities, neck	8	Mother	–	TRB 250 mg/d for 2 m	FLZ 200 mg/d for 2 m with RES	TmVIII/MI1264	Phe397Leu
47	F	49	Upper extremities, trunk-front	12	Child	–	TRB 250 mg/d for 1 m	FLZ 200 mg/d for 2 m with RES	TmVIII/MI1265	Phe397Leu
48	F	22	Lower extremities, groin and gluteal regions	14	Partner	–	TRB 250 mg/d for 1 m	ITZ 100 mg/d for 2 m	TmVIII/MI1271	Phe397Leu
49	F	51	Trunk-front and back, upper and lower extremities, groin and gluteal regions	12	Parents and sibling	–	TRB 250 mg/d for 1 m	FLZ 200 mg/d for 2 m with RES	TmVIII/MI1272	Phe397Leu Ala448Thr
50	M	30	Trunk-front, face, groin and gluteal regions, upper extremities^g^	6	Parents and sibling	–	FLZ 150 mg/w for 1 m, TRB for 1 m, ITZ 100 mg/day for 1 m	ITZ 100 mg/d for 2 m	TmVIII/MI1273	Phe397Leu
51	M	17	Groin and gluteal regions	7	–	–	TRB 250 mg/d for 1 m	FLZ 200 mg/d for 2 m	TmVIII/MI1275	Phe397Leu
52	M	21	Groin and gluteal regions, face	8	–	–	TRB 250 mg/day for 1 m, ITZ 100 mg/d for 2 m	FLZ 200 mg/d for 2 m with RES	TmVIII/MI1276	Phe397Leu
53	F	44	Trunk-front and back, lower extremities, groin and gluteal regions, neck	7	Partner	Diabetes mellitus, hypertension	TRB 250 mg/day for < 1 m, ITZ 100 mg/d for 1 m	FLZ 200 mg/d for 2 m with RES	TmVIII/MI1281	Phe397Leu Ala448Thr
54	F	32	Lower extremities, groin and gluteal regions	8	–	–	TRB 250 mg/d for 1 m, ITZ 100 mg/d for 3 m, FLZ 200 mg/w for 1 m	FLZ 200 mg/d for 4 m with RES partial progress	TmVIII/MI1282	Phe397Leu Ala448Thr
55	F	32	Face, groin and gluteal regions, upper and lower extremities, trunk-back, neck	6	–	Allergic asthma	TRB 250 mg/d for 1 m, FLZ 200 mg/d for 1 m, ITZ 100 mg/d for 7 m, ITZ 200 mg/d 2 m	ITZ 200 mg/d for 3 m, partial progress	TmVIII/MI1284	Phe397Leu Ala448Thr
56	F	40	Groin and gluteal regions	10	Partner	–	TRB 250 mg/d for 1 m	FLZ 200 mg/d for 2 m with RES	TmVIII/MI1286	Phe397Leu
57	F	16	Trunk-front and back, upper and lower extremities, groin and gluteal regions, face, neck, armpit	9	Parents and sibling	–	TRB 250 mg/d for 1 m	FLZ 200 mg/d for 2 m with RES	TmVIII/MI1287	Phe397Leu
58	F	51	Trunk-front and back, upper and lower extremities, groin and gluteal regions	12	Parents and sibling	–	TRB 250 mg/d for 1 m	FLZ 200 mg/d for 2 m with RES	TmVIII/MI1288	Phe397Leu
59	F	16	Trunk-front and back, upper and lower extremities, groin and gluteal regions, face, neck, armpit	9	Parents and sibling	–	TRB 250 mg/d for 1 m	FLZ 200 mg/d for 2 m with RES	TmVIII/MI1289	Phe397Leu Ala448Thr
60	F	55	Lower extremities	12	–	–	TRB 250 mg/d for 1 m	ITZ 200 mg/d for 2 m	TmVIII/MI1	Phe397Leu
61	F	39	Upper extremities, trunk-front, face, neck	6	–	Diabetes mellitus, hypertension	TRB 250 mg/d for 3 m	VRZ (2 × 200 mg for the first two days, then 200 mg/day for 2 m)	TmVIII/MI3	Phe397Leu Ala448Thr
62	M	25	Lower extremities	9	–	–	TRB 250 mg/d for 2 m	ITZ 200 mg/d for 2 m	TmVIII/MI5	Phe397Leu
63	M	27	Lower extremities, face, neck	11	–	–	TRB 250 mg/d for 3 m	ITZ 200 mg/d for 2 m	TmVIII/MI6	Phe397Leu
64	F	38	Lower extremities, trunk-front	12	–	Transplant patient	TRB 250 mg/d for 2.5 m	ITZ 200 mg/d for 2 m	TmVIII/MI7	Phe397Leu
65	F	53	Lower extremities, trunk-back	7	–	–	TRB 250 mg/d for 1 m, FLZ 150 mg/w for 1 m, ITZ 100 mg/d for 3 m	ITZ 200 mg/d for 2 m	TmVIII/MI9	Phe397Leu
66	M	28	Trunk-front, groin and gluteal regions, face, neck	11	–	–	FLZ 150 mg/w for 2 m	ITZ 200 mg/d for 2 m	TmVIII/MI11	Phe397Leu
67	M	33	Upper and lower extremities, groin and gluteal regions	6	–	–	ITZ 100 mg/d for 2 m	ITZ 200 mg/d for 2 m	TmVIII/MI15	Phe397Leu
68	F	24	Trunk-front and back, upper and lower extremities	18	–	–	TRB 250 mg/d for 2 m, ITZ 100 mg/d for 3 m	ITZ 200 mg/d for 3 m	TmVIII/MI16	Phe397Leu
69	F	57	Lower extremities, groin and gluteal regions	12	–	–	TRB 250 mg/d for 3 m	ITZ 200 mg/d for 2 m	TmVIII/MI17	Phe397Leu Ala448Thr
70	M	46	Lower extremities, groin and gluteal regions	7	–	–	TRB 250 mg/d for 2 m	ITZ 200 mg/d for 2 m	TmVIII/MI18	Phe397Leu Tyr414His
71	M	40	Upper extremities, trunk-front and back	6	–	–	TRB 250 mg/d for 1 m, FLZ 150 mg/w for 1 m, ITZ 100 mg/d for 2 m	ITZ 200 mg/d for 2 m	TmVIII/MI20	Phe397Leu Ala448Thr
72	M	54	Lower extremities, groin and gluteal regions, trunk-front, armpit	6	–	–	TRB 250 mg/d for 2 m, FLZ 150 mg/w for 1 m	ITZ 200 mg/d for 2 m	TmVIII/MI21	Phe397Leu
73	F	43	Upper and lower extremities, trunk-front and back, groin and gluteal regions	9	–	–	FLZ 150 mg/w for 2 m	TRB 250 mg/d for 3 m	TmVIII/MI22	Phe397Leu
74	F	39	Upper and lower extremities, trunk-front, groin and gluteal regions	12	–	–	FLZ 150 mg/w for 1 m	ITZ 200 mg/d for 3 m	TmVIII/MI23	Leu393Ser
75	F	47	Trunk-front, groin and gluteal regions	18	–	–	TRB 250 mg/d for 3 m	TRB 250 mg/d for 2 m	TmVIII/MI25	Phe397Leu
77	M	51	Lower extremities, trunk-front	7	–	–	TRB 250 mg/d for 2 m	ITZ 200 mg/d for 2 m	TmVIII/MI27	Phe397Leu Tyr414His
78	M	32	Lower extremities, groin and gluteal regions	6	–	–	TRB 250 mg/d for 2 m, ITZ 100 mg/day for 2 m	FLZ 200 mg/d for 2 m	*T*. *rubrum*/MI1185	*T*. *rubrum* WT
79	M	63	Upper and lower extremities	24	–	–	TRB 250 mg/d, ITZ 200 mg/d for 3 m	ITZ 200 mg/d for 2 m	*T*. *rubrum*/MI1216	Leu393Ser
80	M	27	Trunk-front, groin and gluteal regions	8	–	–	TRB 250 mg/d for 4 m	ITZ 200 mg/d for 2 m	*T*. *rubrum*/MI1218	*T*. *rubrum* WT
81	M	19	Lower extremities, groin and gluteal regions, armpit	7	F	–	TRB 250 mg/d for 2 m	FLZ 200 mg/d for 2 m	*T*. *rubrum*/MI1224	*T*. *rubrum* WT
82	F	69	Trunk-front and back, groin and gluteal regions	6	C	Hypertension	TRB 250 mg/d for 2 m	ITZ 100 mg/d for 2 m	*T*. *rubrum*/MI1242	*T*. *rubrum* WT
83	F	59	Upper and lower extremities, trunk-front, groin and gluteal regions	8	–	Asthma, Diabetes mellitus	TRB 250 mg/d for 2 m, ITZ 200 mg/d for 2 m	FLZ 200 mg/d for 2 m	*T*. *rubrum*/MI1266	**Pro423Ser**
84	F	34	Upper and lower extremities, trunk-front	9	–	–	TRB 250 mg/d for 2 m	ITZ 200 mg/d for 2 m	*T*. *rubrum*/MI2	**Ala445Gly**
85	F	49	Upper and lower extremities, trunk-front	7	–	–	None	TRB 250 mg/d for 2 m	*T*. *rubrum*/MI4	*T*. *rubrum* WT
86	M	41	Groin and gluteal regions	14	–	–	None	TRB 250 mg/d for 2 m	*T*. *rubrum*/MI10	*T*. *rubrum* WT
87	F	37	Feet	12	–	–	None	ITZ 200 mg/d for 2 m	*T*. *rubrum*/MI12	*T*. *rubrum* WT
88	F	25	Lower extremities, trunk-back	10	–	–	TRB 250 mg/d for 2 m	TRB 250 mg/d for 2 m	*T*. *rubrum*/MI13	*T*. *rubrum* WT
89	M	26	Lower extremities, trunk-front, groin and gluteal regions	11	–	–	TRB 250 mg/d for 2 m	ITZ 200 mg/d for 2 m	*T*. *rubrum*/MI24	*T*. *rubrum* WT
76	M	32	Upper extremities, feet^f^	14	–	–	None	ITZ 200 mg/d for 2 m	*T*. *rubrum*/MI26	*T*. *rubrum* WT
90	M	49	Trunk-front and back, groin and gluteal regions	16	–	–	TRB 250 mg/d for 2 m, ITZ 100 mg/d for 2 m	ITZ 200 mg/d for 2 m	*T*. *tonsurans* MI1198	No WT data available for comparison
91	F	33	Groin and gluteal regions	9	–	–	TRB 250 mg/d for 2 m	TRB 250 mg/d for 2 m	*Microsporum canis*/MI19	No data

The amino acid substitutions detected for the first time in this study are shown in bold

*CUR* curcumin, *F* female, *FLZ* fluconazole, *ITZ* itraconazole, *M* male, *MI* Macit Ilkit culture collection, *RES* resveratrol, *T Trichophyton*, *TmVIII Trichophyton mentagrophytes* ITS genotype VIII (*T*. *indotineae*), *TRB* terbinafine, *VRZ* voriconazole, *WT* wild type

^a^*d* day, *m* month, *w* week, *y* year

^b^*Ala* alanine, *Cys* cystein, *Gly* glycine, *His* histidine, *Leu* leucine, *Phe* phenylalanine, *Pro* proline, *Ser* serine, *Thr* threonine, *Tyr* tyrosine, *Val* valine

^c^Full recovery during the treatment, recurrence when the treatment stops

^d^Short-term recovery was achieved. Seizures were reported in this patient due to fluconazole and terbinafine

^e^Maculopapular type id reaction was observed

^f^Itchiness was not reported

^g^Dyshidrotic type id reaction was observed

Among the 13 *T*. *rubrum* isolates, ten were identical to the wild-type *T*. *rubrum* reference strain (DK-Trub-sqle-WT; GenBank accession number OM313296.1), whereas three carried various substitutions in their *SQLE* gene, namely Ala445Gly, Leu393Ser, and Pro423Ser (Table 1). The only hitherto available *T*. *tonsurans SQLE* sequence in GenBank is the one reported by Salehi et al. [36] from a TRB-resistant strain in Iran (GenBank accession number MH523151.1). Our *SQLE* sequence from isolate MI1198 and the Iranian sequence differed by two bases; however, the short Iranian sequence (only 521 bp) limits the detection of additional substitutions potentially involved in TRB resistance. The partial *SQLE* sequence for the *M*. *canis* isolate could not be obtained.

Phylogenetic trees were constructed using ITS, *tubb*, and *tef-1α* sequences from our isolates, together with the ex-type strains of each species and variety. Based on the BIC, the best-fit models were TN + F + G4 for ITS (129 sequences, 608 bp, 152 parsimony-informative sites, and 442 constant sites), K2P for *tubb* (102 sequences, 392 bp, 80 parsimony-informative sites, and 309 constant sites), and TNe + I + F for *tef-1α* (109 sequences, 605 bp, 140 parsimony-informative sites, and 459 constant sites) loci.

### Antifungal Susceptibility Testing

The in vitro antifungal susceptibility test results are summarized in Table 2. Briefly, the MIC_50_ values for FLZ were ≥ 16 mg/L in 71 isolates, consisting of 61 TmVIII, eight *T*. *rubrum*, one *T*. *tonsurans*, and one *M*. *canis*. Of these, 17 isolates (15 TmVIII) had MIC values ≥ 64 mg/L. Since no clinical breakpoints are available for dermatophytes, ITZ, TRB, and VRZ resistance were defined according to the EUCAST-tentative epidemiological cut-off values (TECOFF) [37]. MIC values for ITZ were ≥ 0.25 for six TmVIII (8.2%) and one *T*. *rubrum* isolates, suggesting reduced in vitro activity against the tested dermatophytes. TRB resistance was evaluated using TECOFF values of 0.03 mg/L for *T*. *rubrum* and 0.125 mg/L for *T*. *indotineae*. Overall, 72 TmVIII isolates (98.6%) and two *T*. *rubrum* isolates demonstrated non-WT profiles. Among those, 52 TmVIII isolates presented MICs between 0.25 and 2 mg/L, and 20 isolates had higher MICs (MICs ≥ 4 mg/L). VRZ activity was evaluated using TECOFF values 0.125 mg/L for *T*. *rubrum* and 1 mg/L for *T*. *indotineae* [37]. All *T*. *rubrum* isolates shared the WT-profile, while three TmVIII (4.1%) had MICs of 2 mg/L. Complete inhibition values for resveratrol ranged from 4 to 256 mg/L for TmVIII and from 8 to 64 mg/L for *T*. *rubrum* isolates. The MIC_50_ ranges for each antifungal agent, as well as the complete inhibition ranges for RES, are listed in Table 3. In this study, the results of the CUR activity were provided solely based on clinical response because its poor solubility during analysis precluded the reliable determination of MIC.

**Table 2:** Antifungal susceptibility test results combined with the *SQLE* substitutions of the isolates

Isolate	Species ID	MIC 50 (mg/L)						TGI (mg/L)	*SQLE*
		FLZ	TRB	ITZ	PCZ	VRZ	KCZ	RES	Substitution
MI 1177	TmVIII (*T*. *indotineae*)	32	2	0.016	0.016	0.25	0.125	32	Phe397Leu
MI 1179	TmVIII	32	2	0.064	0.064	0.5	0.25	64	Phe397Leu **Phe446Leu** Ala448Thr
MI 1182	TmVIII	16	2	0.016	0.016	0.125	0.125	32	Phe397Leu
MI 1186	TmVIII	4	2	≤ 0,008	≤ 0,008	0.064	0.064	8	Phe397Leu Tyr414His
MI 1194	TmVIII	16	2	0.016	0.016	0.125	0.064	8	Phe397Leu
MI 1195	TmVIII	16	2	≤ 0,008	≤ 0,008	0.125	0.064	8	Phe397Leu
MI 1196	TmVIII	64	4	0.125	0.064	1	0.5	64	Phe397Leu Ala448Thr
MI 1197	TmVIII	32	0.008	0.032	0.064	0.5	0.25	32	**Phe446Leu** Ala448Thr
MI 1199	TmVIII	16	1	0.016	0.016	0.125	0.125	32	Phe397Leu
MI 1201	TmVIII	8	> 4	≤ 0,008	0.016	0.125	0.064	8	Phe397Leu Tyr414His
MI 1204	TmVIII	32	> 4	0.064	0.064	0.25	0.5	128	Phe397Leu
MI 1206	TmVIII	16	> 4	0.032	0.032	0.25	0.125	32	Phe397Leu **Phe446Gly** Ala448Thr
MI 1207	TmVIII	32	2	0.032	0.032	0.25	0.125	32	Phe397Leu
MI 1208	TmVIII	64	2	0.5	0.125	1	0.5	128	Phe397Leu Ala448Thr
MI 1210	TmVIII	16	2	≤ 0,008	0.016	0.125	0.125	8	Phe397Leu
MI 1211	TmVIII	32	2	0.032	0.032	0.25	0.125	64	Phe397Leu
MI 1212A	TmVIII	16	> 4	0.016	0.016	0.125	0.125	16	Phe397Leu
MI 1212B	TmVIII	> 64	> 4	0.5	0.25	2	1	256	Phe397Leu
MI 1213	TmVIII	4	4	≤ 0,008	0.016	0.064	0.064	8	Phe397Leu Tyr414His
MI 1214	TmVIII	64	2	0.064	0.125	1	0.5	128	Phe397Leu **Phe446Cys** Ala448Thr
MI 1215	TmVIII	16	1	0.016	0.016	0.125	0.064	256	Phe397Leu **Phe446Val**
MI 1219	TmVIII	32	2	0.064	0.064	0.5	0.25	64	Phe397Leu **Phe446Leu** Ala448Thr
MI 1195–2	TmVIII	16	1	≤ 0,008	≤ 0,008	0.064	0.064	16	Phe397Leu
MI 1221	TmVIII	ND	ND	ND	ND	ND	ND	ND	Phe397Leu
MI 1222	TmVIII	16	2	≤ 0,008	≤ 0,008	0.125	0.064	32	Phe397Leu **Val436Gly**
MI 1225	TmVIII	16	> 4	≤ 0,008	≤ 0,008	0.064	0.064	8	Phe397Leu Tyr414His
MI 1226	TmVIII	ND	ND	ND	ND	ND	ND	ND	Phe397Leu
MI 1227	TmVIII	8	2	≤ 0,008	0.016	0.064	0.125	16	Phe397Leu
MI 1230	TmVIII	64	2	0.032	0.125	2	0.5	64	Phe397Leu Ala448Thr
MI 1231	TmVIII	32	2	0.032	0.064	0.5	0.25	128	Phe397Leu Ala448Thr
MI 1232	TmVIII	8	> 4	0.032	0.016	0.125	0.125	16	Phe397Leu Tyr414His
MI 1234	TmVIII	16	2	0.032	0.032	0.25	0.125	32	Phe397Leu
MI 1236	TmVIII	32	2	0.032	0.032	0.5	0.25	64	Phe397Leu Ala448Thr
MI 1244	TmVIII	8	4	≤ 0,008	≤ 0,008	0.064	0.064	8	Phe397Leu Tyr414His
MI 1247	TmVIII	16	2	≤ 0,008	≤ 0,008	0.125	0.125	16	Phe397Leu
MI 1249	TmVIII	> 64	2	8	0.125	1	0.5	128	Phe397Leu Ala448Thr
MI 1252	TmVIII	8	2	≤ 0,008	≤ 0,008	0.064	0.064	16	Phe397Leu
MI 1253	TmVIII	64	> 4	0.125	0.125	0.25	0.5	128	Phe397Leu
MI 1254	TmVIII	16	1	≤ 0,008	0.016	0.064	0.125	32	Phe397Leu
MI 1255	TmVIII	16	2	≤ 0,008	≤ 0,008	0.064	0.064	16	Phe397Leu
MI 1257	TmVIII	ND	ND	ND	ND	ND	ND	ND	Phe397Leu
MI 1259	TmVIII	32	2	0.016	0.016	0.125	0.125	16	Phe397Leu
MI 1261	TmVIII	8	1	≤ 0,008	≤ 0,008	0.064	0.125	32	Phe397Leu
MI 1262	TmVIII	8	> 4	≤ 0,008	≤ 0,008	0.064	0.125	8	Phe397Leu Tyr414His
MI 1263	TmVIII	16	1	≤ 0,008	0.016	0.125	0.125	128	Phe397Leu
MI 1264	TmVIII	32	2	0.016	0.016	0.25	0.125	32	Phe397Leu
MI 1265	TmVIII	32	2	0.016	0.016	0.25	0.125	128	Phe397Leu
MI 1271	TmVIII	32	1	0.032	0.016	0.25	0.125	32	Phe397Leu
MI 1272	TmVIII	64	4	0.125	0.125	0.5	0.5	64	Phe397Leu Ala448Thr
MI 1273	TmVIII	16	2	0.016	0.016	0.125	0.125	16	Phe397Leu
MI 1275	TmVIII	16	2	≤ 0,008	≤ 0,008	0.125	0.125	32	Phe397Leu
MI 1276	TmVIII	16	2	≤ 0,008	0.016	0.064	0.125	8	Phe397Leu
MI 1281	TmVIII	32	2	0.5	0.125	1	0.5	128	Phe397Leu Ala448Thr
MI 1282	TmVIII	64	2	2	1	1	2	64	Phe397Leu Ala448Thr
MI 1284	TmVIII	32	1	0.032	0.032	0.25	0.25	32	Phe397Leu Ala448Thr
MI 1286	TmVIII	16	1	0.016	0.016	0.125	0.125	32	Phe397Leu
MI 1287	TmVIII	16	2	0.016	0.016	0.125	0.064	16	Phe397Leu
MI 1288	TmVIII	4	0.5	≤ 0,008	≤ 0,008	0.016	0.016	4	Phe397Leu
MI 1289	TmVIII	32	4	0.125	0.125	0.5	0.5	64	Phe397Leu Ala448Thr
MI 1	TmVIII	> 64	2	0.064	0.125	1	1	64	Phe397Leu
MI 3	TmVIII	> 64	4	2	0.25	2	1	256	Phe397Leu Ala448Thr
MI 5	TmVIII	32	2	0.016	0.032	0.25	0.125	16	Phe397Leu
MI 6	TmVIII	32	2	0.016	0.016	0.25	0.125	32	Phe397Leu
MI 7	TmVIII	32	2	0.032	0.032	0.25	0.125	128	Phe397Leu
MI 9	TmVIII	16	2	0.016	0.032	0.125	0.125	16	Phe397Leu
MI 11	TmVIII	32	1	0.016	0.016	0.125	0.125	32	Phe397Leu
MI 15	TmVIII	8	2	≤ 0,008	≤ 0,008	0.125	0.032	64	Phe397Leu
MI 16	TmVIII	16	1	0.016	0.016	0.125	0.125	32	Phe397Leu
MI 17	TmVIII	64	1	0.064	0.125	1	0.5	256	Phe397Leu Ala448Thr
MI 18	TmVIII	8	> 4	0.016	≤ 0,008	0.064	0.064	8	Phe397Leu Tyr414His
MI 20	TmVIII	64	4	0.125	0.125	1	0.5	64	Phe397Leu Ala448Thr
MI 21	TmVIII	16	1	≤ 0,008	0.016	0.064	0.125	32	Phe397Leu
MI 22	TmVIII	64	> 4	0.125	0.125	0.5	1	128	Phe397Leu
MI 23	TmVIII	> 64	0.25	0.016	0.064	0.5	2	32	Leu393Ser
MI 25	TmVIII	32	4	0.032	0.032	0.25	0.125	32	Phe397Leu
MI 27	TmVIII	16	4	≤ 0,008	≤ 0,008	0.125	0.064	8	Phe397Leu Tyr414His
MI 26	*T*. *rubrum*	32	≤ 0,004	0.016	0.032	0.064	0.5	64	*T*. *rubrum* WT
MI 1185	*T*.* rubrum*	32	≤ 0,004	0.032	0.032	0.064	0.5	32	*T*. *rubrum* WT
MI 1216	*T*.* rubrum*	8	0.064	0.032	0.032	0.125	0.25	32	Leu393Ser
MI 1218	*T*.* rubrum*	2	≤ 0,004	0.016	0.016	0.032	0.032	16	*T*. *rubrum* WT
MD1224	*T*.* rubrum*	8	≤ 0,004	0.016	0.016	0.064	0.125	16	*T*. *rubrum* WT
MD1242	*T*.* rubrum*	ND	ND	ND	ND	ND	ND	ND	*T*. *rubrum* WT
MI 1266	*T*.* rubrum*	4	0.008	≤ 0,008	≤ 0,008	0.032	0.064	8	**Pro423Ser**
MI 2	*T*.* rubrum*	16	≤ 0,004	0.016	0.032	0.032	0.25	16	**Ala445Gly**
MI 4	*T*.* rubrum*	16	≤ 0,004	0.016	0.032	0.032	0.25	32	*T*. *rubrum* WT
MI 10	*T*.* rubrum*	32	≤ 0,004	0.064	0.125	0.064	1	64	*T*. *rubrum* WT
MI 12	*T*.* rubrum*	16	≤ 0,004	0.016	0.016	0.032	0.25	16	*T*. *rubrum* WT
MI 13	*T*.* rubrum*	> 64	> 4	> 8	≤ 0,008	0.016	0.125	16	*T*. *rubrum* WT
MI 24	*T*.* rubrum*	32	≤ 0,004	0.064	0.064	0.125	1	32	*T*. *rubrum* WT
MI 1198	*T*.* tonsurans*	32	0.008	0.032	0.064	0.064	0.25	64	No WT data available for comparison
MI 19	*M*. *canis*	64	0.008	4	0.25	0.25	1	128	No data

*CUR* curcumin, *FLZ* fluconazole, *ITZ* itraconazole, *KCZ* ketoconazole, *MI* Macit Ilkit culture collection, *MIC* minimum inhibitory concentration, *PCZ* posaconazole, *RES* resveratrol, *T Trichophyton*, *TGI* total growth inhibition concentration, *TRB* terbinafine, *TmVIII T*. *mentagrophytes* ITS genotype VIII, *VRZ* voriconazole, *WT* wild type. The amino acid substitutions detected for the first time in this study are shown in bold

**Table 3:** The minimum inhibitory concentration ranges for FLZ, ITZ, KCZ, PCZ, TRB, and VRZ, and total growth inhibition concentration ranges for RES, against the *Trichophyton mentagrophytes* ITS genotype VIII (*T. indotineae*) and *T. rubrum* isolates

Isolates	Antifungal drug	MIC 50 range (mg/L)	TGI range (mg/L)	GM (mg/L)
*Trichophyton mentagrophytes* ITS genotype VIII (*n* = 73)	FLZ	4–64		22.52
	ITZ	0.008–8		0.005
	KTZ	0.016–2		0.17
	PCZ	0.008–1		0.04
	TRB	0.008–4		1.89
	VRZ	0.016–2		0.21
	RES		4–256	34.2
*Trichophyton rubrum* (*n* = 12)	FLZ	2–64		15.1
	ITZ	0.008–8		0.02
	KCZ	0.032–1		0.24
	PCZ	0.008–0.125		0.03
	TRB	0.004–4		0.009
	VRZ	0.016–0.125		0.05
	RES		8–64	24

*TGI* total growth inhibition concentration, *FLZ* fluconazole, *GM* geometric means, *ITZ* itraconazole, *KCZ* ketoconazole, *MIC* minimum inhibitory concentration, *PCZ* posaconazole, *RES* resveratrol, *TRB* terbinafine, *VRZ* voriconazole

### Clinical Response to Antifungal Treatments

Successful treatment was defined as complete lesion clearance and negative KOH microscopy. When there was no improvement of the lesions after 4 weeks, or erythema and/or scales were observed at 8 weeks, and microscopy analysis revealed positive results for fungal elements at these follow-ups, the cases were considered failed. If the erythema and/or scales were observed at the site of healed lesions during the follow-up of healed patients (at 8 weeks), these cases were considered as recurrence.

All patients had previously received topical and/or systemic antifungal therapy as monotherapy or in combination without clinical improvement. These failed regimens included TRB alone (*n* = 49), TRB + ITZ (*n* = 18), TRB + ITZ + FLZ (*n* = 10), ITZ alone (*n* = 7), TRB + FLZ (*n* = 6), and FLZ + ITZ (*n* = 1). During the study, 83/91 patients responded to the different treatment regimens (in terms of their doses, durations, or combinations) with FLZ (200 mg/d), ITZ (100‒200 mg/d), TRB (250 mg/d), or VRZ (200 mg twice daily for 2 days, followed by 200 mg/d for 8 weeks) (Table 1). The most commonly used antifungals were ITZ (*n* = 41) and FLZ (*n* = 36). Among patients with previous TRB failure (*n* = 83), ITZ and FLZ monotherapy provided clinical improvement in 36/83 (43.4%) and 33/83 (39.8%), respectively. Among 41 patients who received ITZ, three were successfully treated with ITZ (200 mg/d) in combination with CUR supplement (40 mg/d for 1 month) and the overall success rate of ITZ was 93%. In three other patients (isolates MI1195, MI1201, and MI1284), ITZ monotherapy (200 mg/d for 2 months) initially cleared lesions, but recurrence occurred shortly after treatment discontinuation. One patient (isolate MI1247) was successfully treated with ITZ (100 mg/d) in combination with topical sertaconazole, remaining 90 patients used luliconazole cream as a topical treatment. Thirty-five patients received FLZ therapy and 33 of them fully recovered; 25 of them were a combination of FLZ (200 mg/d for 1–4 months) and RES (2 × 200 mg/d for 1–4 months), and only one patient did not respond to this treatment. The overall success rate of FLZ was 94%. In 12 patients, lesions were resolved; however, post-inflammatory hyperpigmentation persisted. Recurrence and partial improvement of the lesions were observed in two (2.2%) and three patients (3.3%), respectively. Detailed information on the dosage and duration of both the most recent therapy applied before the study, and successful treatments is provided in Table 1.

An adverse event was observed in one patient receiving phenobarbital for epilepsy who developed seizures during both TRB and FLZ therapy, prompting discontinuation. This patient tolerated ITZ (200 mg/d), although only partial clinical remission was observed. Seven patients did not respond to TRB, ITZ, or FLZ (in combination with RES) treatments, despite the absence of drug-drug interactions.

The combined analysis of clinical outcomes, in vitro TRB susceptibility, and *SQLE* substitution profiles revealed several discordant findings. The patient from whom the MI1199 isolate (harboring the Phe378Leu *SQLE* substitution) was obtained had previously received combined TRB (250 mg/d) and ITZ (200 mg/d) therapy for 3 months without improvement. Surprisingly, the same patient achieved complete clinical remission with FLZ (200 mg/d for 2 months) despite in vitro MIC values of 16 mg/L and 1 mg/L for FLZ and TRB, respectively. Similarly, a combination of FLZ (200 mg/d) and RES (2 × 200 mg) was effective in 14/29 patients even though their isolates exhibited high MICs for FLZ (32 to ≥ 64 mg/L).

## Discussion

Dermatophytosis has long been considered a neglected fungal infection, as it is not considered life-threatening, and has usually been straightforward to treat. However, the emergence of recalcitrant cases and antifungal resistance has complicated management in recent years. Easy access to over-the-counter topical antifungal-corticosteroid combinations likely contributes to selective pressure and rising resistance among dermatophytes [38]. Environmental factors (e.g., higher ambient temperatures and humidity) and social factors (e.g., crowded living conditions, poor hygiene, or sharing utensils within large households) further facilitate the survival and transmission of dermatophytes, complicating surveillance and control efforts. In the current study, more than one-third of the participants reported similar symptoms among their family members (Table 1). Therefore, where possible, all infected family members were invited for clinical evaluation and were followed throughout their treatment course to prevent reinfection and ongoing transmission. On the other hand, it was impossible to identify the source of the infection when there was no connection with any known sources of dermatophytosis, and if the patients were working or living in circumstances where they were constantly exposed to various possible sources of infection (for instance, as a sex worker or a prisoner).

Following the initial description in India [39], similar cases have been reported in several other countries [8]. The first such cases in Türkiye were reported in 2023 [17]. Although numerous studies have examined dermatophyte prevalence in Türkiye over the years [41–45], no retrospective study has re-examined the presence of TmVIII among previously identified *T*. *mentagrophytes* isolates. Consequently, whether resistant TmVIII strains were circulating in the country before 2023 remains unknown. In addition, 83 of 91 patients in the present study had prior TRB exposure (monotherapy or in combination), meaning that the clinical samples were collected after TRB therapy. Accordingly, we could not determine whether TRB resistance was primary or acquired during treatment.

**Fig. 4:**
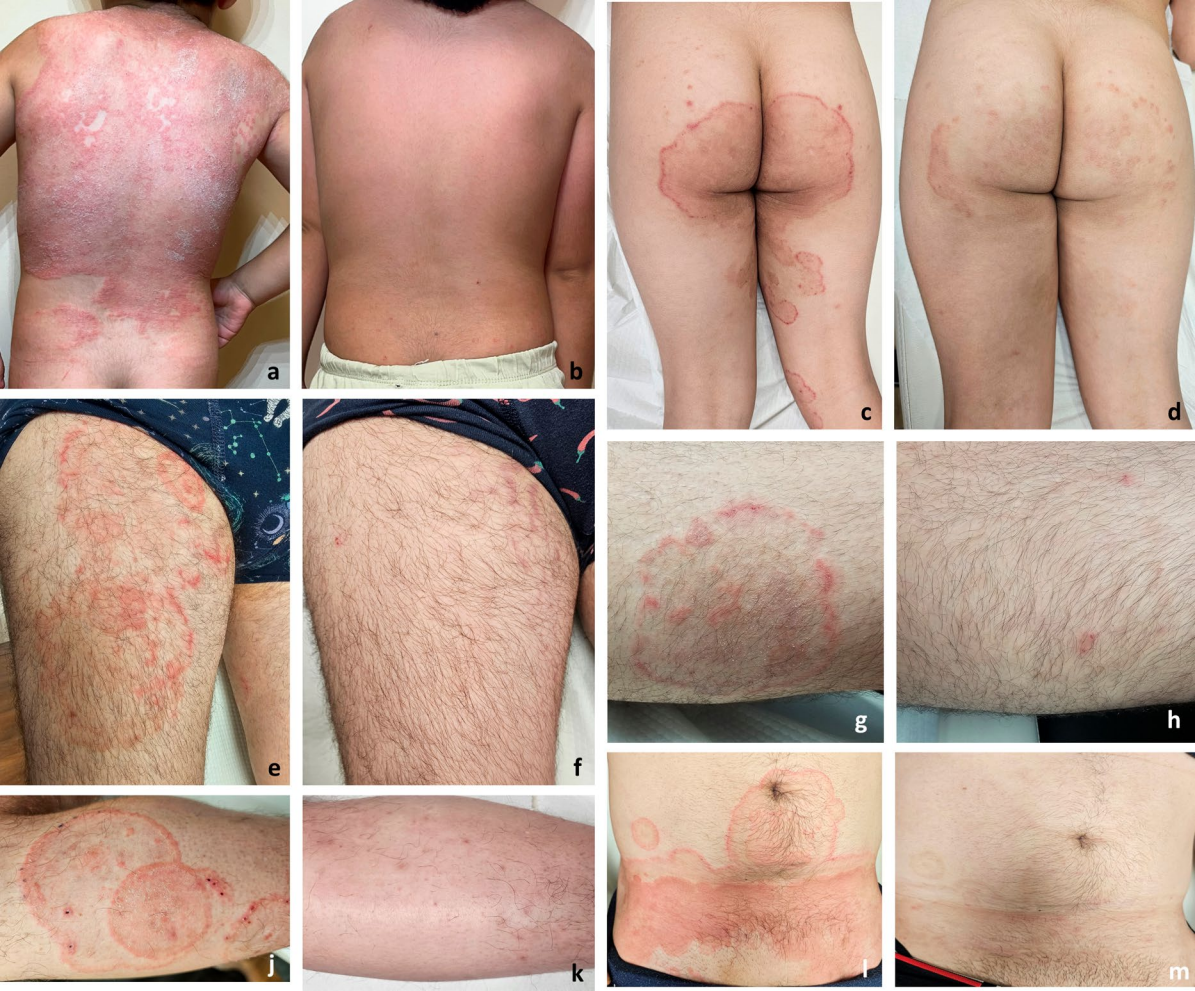
Clinical appearance of the lesions before and after antifungal treatment in different patients. **a**,** b** A 5-year-old male patient (isolate MI1253, TmVIII), **a** erythematous scaly plaques on the back before treatment. **b** after ITZ treatment for 2 months. **c**,** d** A 18-year-old female patient (isolate MI1219, TmVIII) **c** annular erythematous scaly plaques in the gluteal region and thighs, **d** response to FLZ (200 mg/d) treatment for 2 months with post-inflammatory hyperpigmentation. **e–h** A 32-year-old male patient (isolate MI1185, *T*. *rubrum*), erythematous scaly plaques before the treatment, **e** on legs, **g** on the anterior thigh; **f**,** h** the response after FLZ (200 mg/d) treatment for 2 months. **j–m** A 31-year-old male patient (isolate MI1182, TmVIII) annular erythematous scaly plaques on **j** leg, and **l** the lower abdominal quadrant; **k**,** m** response to FLZ (200 mg/d) plus RES treatment for 2 months

TmVIII is usually reported from cases of tinea corporis, tinea cruris, and tinea faciei cases, but has recently also been reported in onychomycosis [6, 46]. This clinical heterogeneity limits the utility of clinical presentation as a taxonomic criterion. The lesions in cases of tinea corporis, tinea cruris, and tinea faciei caused by TmVIII are typically extensive, pruritic, and inflammatory [3] (Fig. 4). Tang et al. [4] proposed that the high virulence of TmVIII might indicate a zoonotic origin for this lineage. Several animal cases have been reported, supporting the hypothesis that animals may act as reservoirs of TmVIII and that zoonotic transmission cannot be excluded [47, 48]. However, in most studies, human cases without known animal contact were prevalent. Additionally, in our cohort, animal contact was not investigated. Nevertheless, it is non-negligible that this lineage might represent a zoophilic ancestor in the process of adaptation to humans. In this case, a decrease in apparent virulence over time might be anticipated, although this remains speculative for the time being.

Several amino acid substitutions in the *SQLE* gene have been associated with TRB resistance in TmVIII and other dermatophyte species. Among them, the Phe397Leu substitution was the most frequently observed [8]. In the current study, in addition to Phe397Leu, we also identified Ala448Thr and Leu393Ser, both of which have been previously reported [24, 35]. Conversely, Tyr414His was detected alongside Phe397Leu in nine TRB-resistant isolates (with MICs ranging from 2 to > 4 µg/mL). To date, Tyr414His has been reported only from England [49], Germany [50], and Türkiye [17].

Although TmVIII commonly exhibits high TRB resistance, in vitro TRB-susceptible isolates have also been reported [51–54]. Among those reports, in one case, clinical treatment with combined oral TRB and topical ketoconazole (KCZ) failed even though the isolate was susceptible to TRB (MIC 0.06 µg/mL) [53]. In our study, only one isolate (MI1197) had a low MIC value (0.008 mg/L) despite the presence of Phe446Leu Ala448Thr substitutions, and the patient of this isolate was successfully treated with TRB. Conversely, in two different patients, TRB treatment was effective despite their isolates displayed high TRB MICs (≥ 4 mg/L) and the Phe397Leu substitution.

In the current study, Ala448Thr substitutions occurred only in combination with other *SQLE* substitutions, except in one case, always found with Phe397Leu. The Ala448Thr substitution has not been shown to confer TRB resistance independently; however, it has been associated with higher MIC values for ITZ and VRZ [35]. In our study, higher MIC values with azoles were not directly linked to Ala448Thr substitution, as other isolates with only Phe397Leu substitutions also showed similar profiles (Table 2). In total, 19 isolates with multiple *SQLE* substitutions that contain Ala448Thr exhibited high MICs for FLZ (ranging from 16 to > 64 mg/L) and TRB MICs (1 to > 4 mg/L). However, 16/19 (84%) were successfully treated with azole-based regimens (Table 1), and 9/16 responded to FLZ therapy combined with RES. There was no significant difference in the success rates of ITZ and FLZ in the treatment of TRB-resistant dermatophyte infections; however, FLZ was slightly more effective than ITZ (94 vs. 93%, respectively), different from the findings reported by Singh et al. [55]. Collectively, these observations indicate that clinical response does not always align with in vitro MICs and *SQLE* genotype alone, implicating that additional factors such as host factors, drug exposure at the infection site, treatment adherence, and further resistance mechanisms may also affect the clinical outcome.

Alternative treatment strategies for dermatophytosis are increasingly considered due to limited access to common antifungals in some countries, the risk of resistance development, or contraindications such as drug–drug interactions. Although VRZ is not a routine first-line treatment nor is it considered standard practice for treating superficial dermatophytoses, in cases where TRB, ITZ, and FLZ treatments have failed, VRZ has been found to be effective [56–58]. Nevertheless, given its importance in the management of life-threatening systemic mycoses, use of VRZ in superficial dermatophytosis should be confined to cases unresponsive to all other antifungals or the presence of drug–drug interactions. Similarly, PCZ is another off-label triazole option and it has previously shown promising results in a multidrug-resistant *T*. *indotineae* infection [9]. KCZ has also been reported as alone or adjunctive topical treatment in TRB-resistant *T*. *indotineae* infections [59]. However, these azoles are currently not recommended as treatment options due to treatment safety and cost concerns [60]. In our study, among these azoles, only VRZ therapy was recommended for seven cases with multidrug resistance; however, these patients were lost to follow-up.

RES has been previously reported as a promising alternative, demonstrating in vitro activity against a broad spectrum of pathogens, including *Aspergillus*, *Candida*, and dermatophytes [13, 61]. In our first report of TRB-resistant TmVIII from Türkiye, a patient with a CD36 mutation was successfully treated with RES tablets without further antifungal therapy [17]. Recently, Kurmus et al. [62] reported a case of TRB-resistant TmVIII that was successfully treated with FLZ at 200 mg/d, oral RES at 200 mg/d, and 1% luliconazole cream over a period of 3 weeks. In the current study, the clinical efficacy of RES was not supported by its in vitro activity against the isolates. Such that, 29 patients received the combination of FLZ and RES, despite the isolates’ elevated in vitro MICs for both agents. All but one patient achieved clinical improvement, and 28 attained complete resolution.

In addition, three patients in this study were successfully treated with ITZ supplemented with CUR. CUR has been previously shown to be effective in vitro against dermatophytes and *Candida* species [63], as well as *Cryptococcus neoformans* and *Paracoccidioides brasiliensis* [64], with reports of enhanced effects when combined with antifungal agents [65]. Despite its in vitro activity against selected fungi and limited clinical efficacy, the application of CUR remains challenging due to its poor oral bioavailability and insufficient solubility in aqueous solvents [66, 67]. Standardized solubilization or formulation approaches and dedicated synergy assays are needed to assess the antifungal activity of CUR against dermatophytes more reliably.

Currently, in vitro discrimination of TRB-resistant TmVIII from susceptible isolates can be performed on SGA plates supplemented with TRB [68, 69]. However, definitive characterization should include the European Committee on Antimicrobial Susceptibility Testing (EUCAST) broth microdilution method to determine MICs (E.Def 11.0) [33] and sequencing of the *SQLE* hotspot region to identify resistance-associated substitutions. While the agar-based screening is simple and useful for surveillance, reporting precise MICs and the underlying *SQLE* substitutions is recommended to track temporal trends in TRB resistance and to detect the regional emergence of specific genotypes.

In conclusion, the cases reported in this study showed that, although the vast majority of TmVIII isolates carried Phe397Leu substitution, a specific Tyr414His substitution is also evident. Considering that 8/9 isolates harboring both Phe397Leu and Tyr414His substitutions showed MIC values ≥ 4 against TRB, Tyr414His might have an impact on TRB resistance in TmVIII. Further studies would reveal whether and how Tyr414His substitution in the *SQLE* gene affects the enzyme structure and functions. In our study group, ITZ-based regimens were the most effective, followed by FLZ-based regimens, in the treatment of TRB-resistant dermatophytosis. Despite efforts to identify TmVIII and treat infections caused by this genotype using certain key features (e.g., affected anatomical regions and high TRB resistance), worldwide reported cases have demonstrated that this genotype shares more similarities with other dermatophytes than previously shown. Reporting both failed and successful treatments, along with the patient and isolate profiles, may increase the chances for other clinicians to follow appropriate regimens earlier when encountering similar cases. Additionally, ongoing molecular surveillance that tracks local MIC distributions and *SQLE* substitution patterns, and compares them across regions, will help us to understand the resistance mechanisms as well as to develop more accurate identification tools and alternative treatments in the future.

## Supplementary Information

Below is the link to the electronic supplementary material.

Supplementary file1 (PDF 1083 KB)

Supplementary file2 (XLSX 49 KB)

Supplementary file3 (XLSX 16 KB)

## Data Availability

All sequencing data generated are available on the NCBI GenBank (Table S2). All phylogenetic trees and alignments are available on Figshare with the identifier number 10.6084/m9.figshare.31084501. Data sets are available upon request to MD and HK.
