# Supplementary material for: Clinical and Mycological Profiles of Chronic and Recalcitrant Dermatophytosis: Türkiye, 2022–2024
Source: Mycopathologia. 2026 Feb 18;191(2):34. doi: 10.1007/s11046-026-01058-5 (PMC12916938; doi:10.1007/s11046-026-01058-5)

Figure S1a Maximum likelihood phylogenetic tree constructed with ITS sequences of the isolates

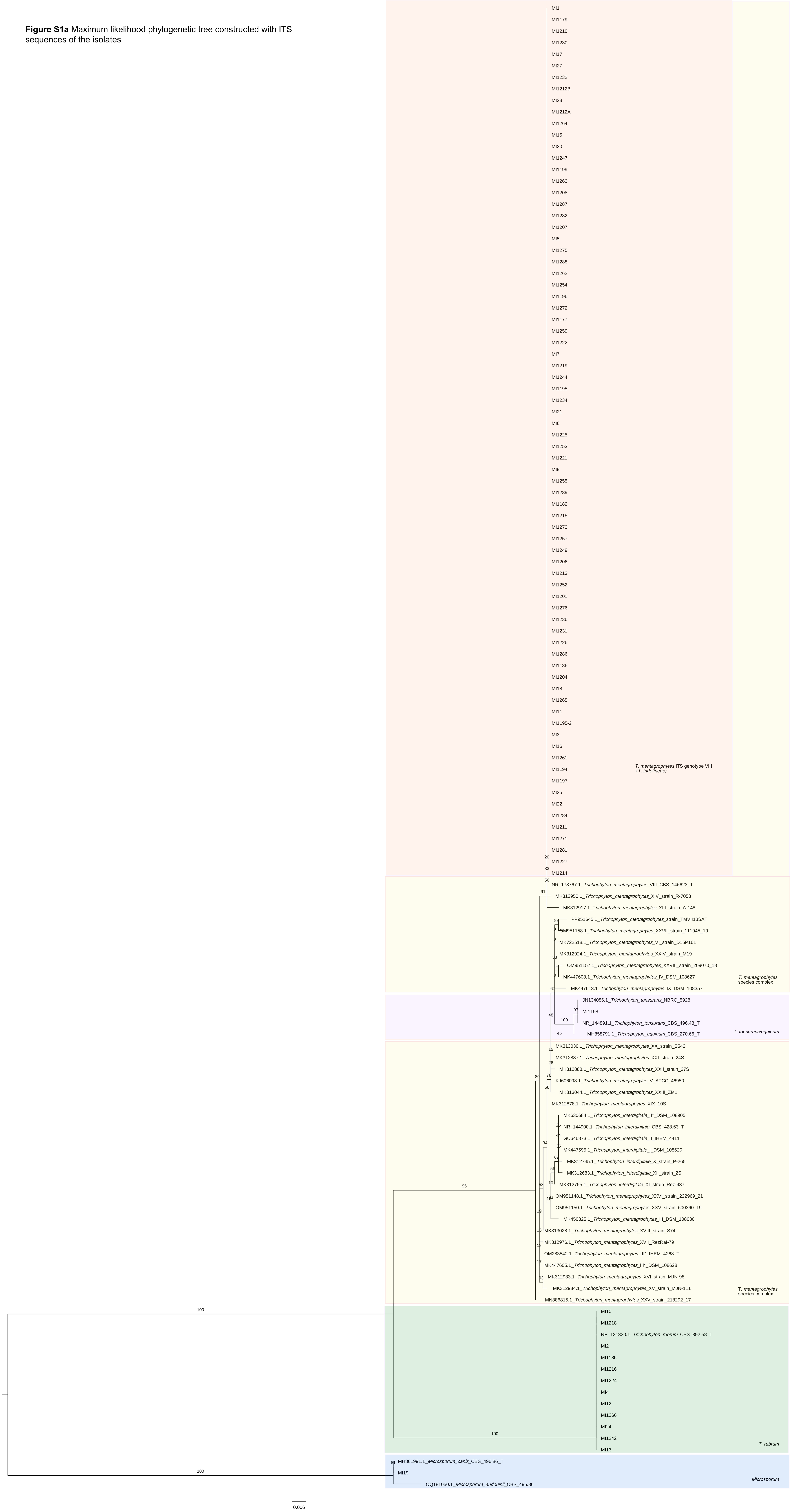

Figure S1b Maximum likelihood phylogenetic tree constructed with *tubb* sequences of the isolates

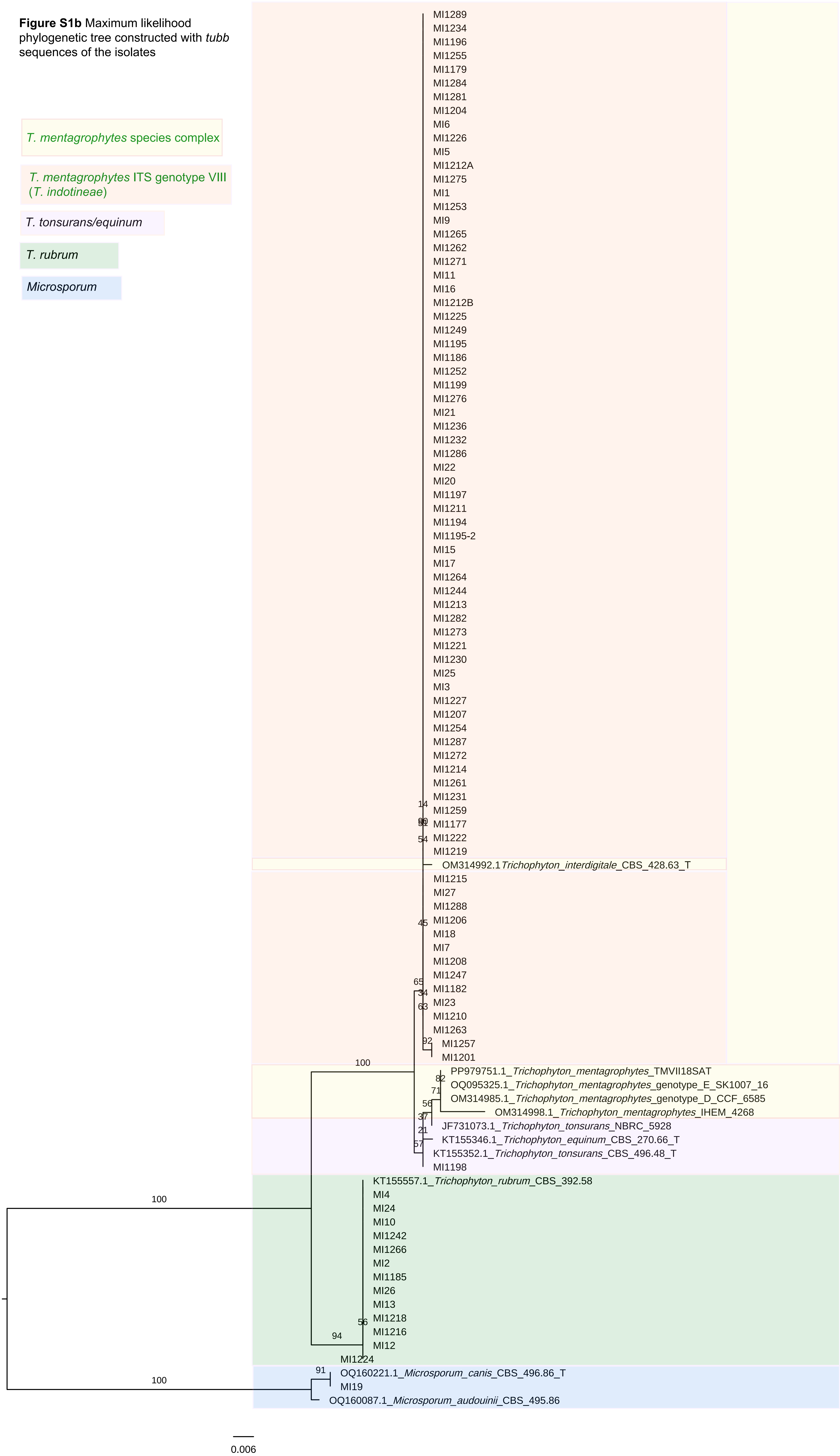

Figure S1c Maximum likelihood phylogenetic tree constructed with *tef-1α* sequences of the isolates

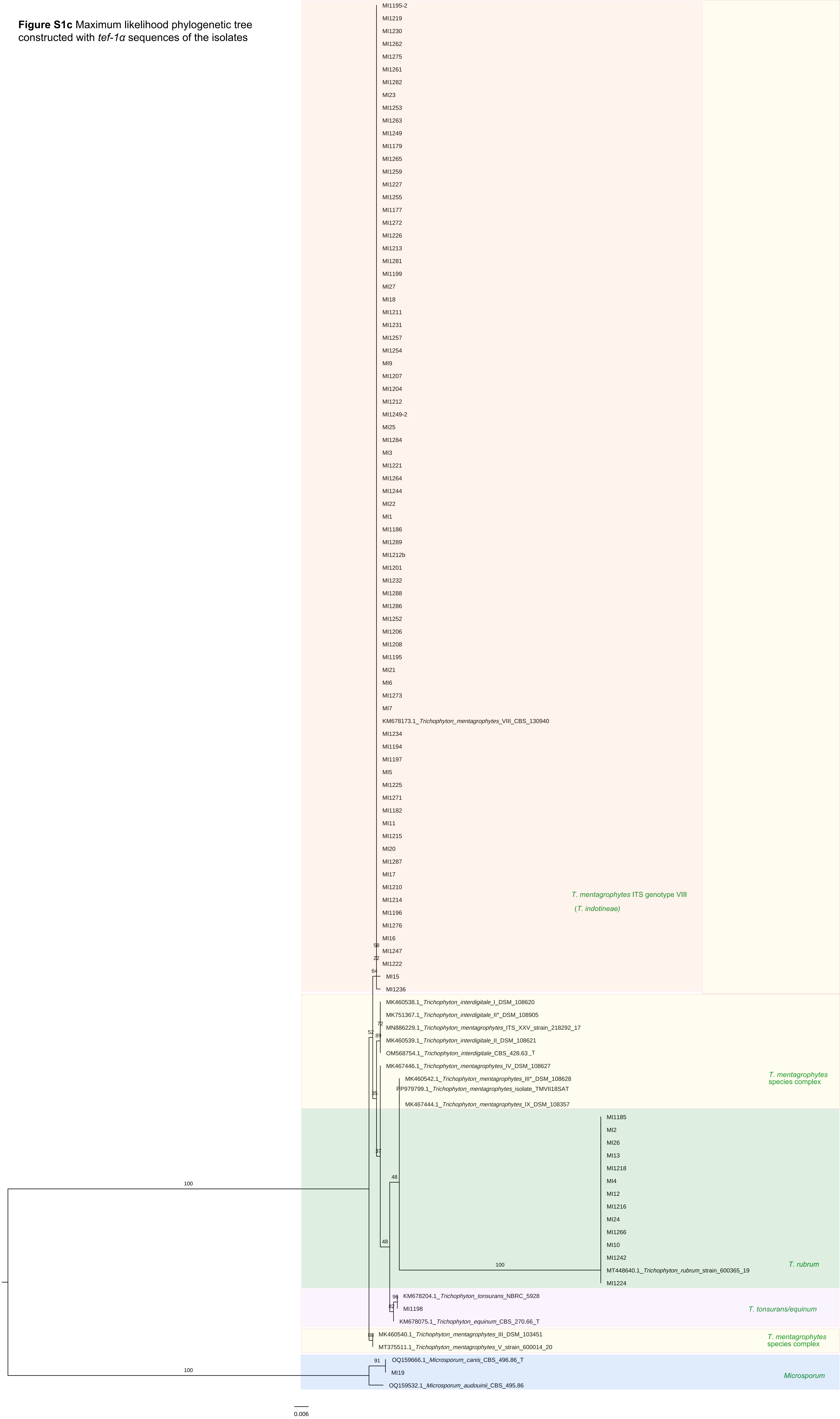

Supplement: Supplementary file 1 — Supplementary file1 (PDF 1083 KB) [file 11046_2026_1058_MOESM1_ESM.pdf]
